# Transient Hyperthyrotropinemia in Outpatient Children with Acute Infections of the Respiratory System

**DOI:** 10.3390/ijerph18084115

**Published:** 2021-04-13

**Authors:** Katarzyna Adamczewska, Zbigniew Adamczewski, Magdalena Stasiak, Andrzej Lewiński, Renata Stawerska

**Affiliations:** 1 Primary Care Unit “Medyk”–Outpatient Clinic, 95-035 Ozorków, Poland; kadamczewska@o2.pl; 2 Department of Nuclear Medicine, Medical University of Lodz, 92-216 Lodz, Poland; zbigniew.adamczewski@umed.lodz.pl; 3 Department of Endocrinology and Metabolic Diseases, Polish Mother’s Memorial Hospital-Research Institute, 93-338 Lodz, Poland; mstasiak33@gmail.com (M.S.); andrzej.lewinski@umed.lodz.pl (A.L.); 4 Department of Endocrinology and Metabolic Diseases, Medical University of Lodz, 93-338 Lodz, Poland; 5 Department of Pediatric Endocrinology, Medical University of Lodz, 93-338 Lodz, Poland

**Keywords:** thyroid stimulating hormone, transient hyperthyrotropinemia, acute respiratory tract infection, children

## Abstract

Background: Diagnostics of thyroid disorders (TD) are frequently based on the measurements of thyroid stimulating hormone (TSH) concentration only. If TSH is outside the reference range, the diagnostic procedure used in patients with TD isintroduced. Observations indicate that in a considerable number of these patients, TD is not confirmed. The aim of the study was to assess the incidence of transient hyperthyrotropinemia in healthy children during acute infections of the respiratory system. Patients and Methods: The study included consecutive children (49 boys and 45 girls), aged 2.2–17.3 years, who visited one General Practitioner (GP) due to respiratory tract infections. The tests: complete blood count (CBC), C-reactive protein (CRP), TSH and FT4 were run on the next day after the visit at the physician’s (initial visit) and ≥2 weeks after recovery. Results: Among these children, elevated TSH values were found in about 10% of patients, and they went back to normal values after recovery. A prospective analysis showed a reduction of TSH values in approx. 65% of all groups and TSH at the follow-up visit was significantly lower. Conclusions: Transient hyperthyrotropinemia was observed in about 10% of children with acute respiratory tract infection. This preliminary finding remains unexplained.

## 1. Introduction

Normal thyroid hormone (thyroxine-T4 and triiodothyronine-T3) secretion and action are essential for both fetal and post-natal neurodevelopment. It is also important for, normal growth and metabolic processes in children [1,2]. The prevalence of thyroid disease in Polish children is not very well known, but in the first two decades after mandatory salt iodination, we observed a lower incidence of goiter and a higher frequency of Graves′ disease [3,4]. In the iodine-sufficient regions, the most common cause of acquired primary hypothyroidism (characterized by low free T4 (FT4) and elevated thyroid stimulating hormone (TSH) serum concentrations) is autoimmune hypothyroidism (Hashimoto’s thyroiditis); its prevalence in childhood has been estimated as 1–2% [1].

In turn, in approximately 2% of children, subclinical hypothyroidism is present. It can be defined as primary biochemically compensated hypothyroidism [1,2]. In these conditions, normal FT4 and free T3 (FT3) and elevated TSH serum concentrations are observed. The treatment of subclinical hypothyroidism is controversial, as it is usually an asymptomatic, benign condition with rare progression to over hypothyroidism. According to the current recommendations, only symptomatic children, children younger than 3 years old, or those with TSH levels higher than 10 mIU/L require the L-T4 treatment [5,6,7]. The etiology of subclinical hypothyroidism is usually idiopathic–especially in the cases of transient hyperthyrotropinemia, but it also may be the initial manifestation of Hashimoto′s thyroiditis [5,6,8].

About 50% of patients with childhood autoimmune hypothyroidism have a family history of such diseases [1]. It is well known that the occurrence of autoimmune thyroid disease (AITD) depends onan interaction between genetic susceptibility and environmental factors. Thus, there is an ongoing debate in scientific associations andthe general public about the factors that can initiate hypothyroidism. As a result, parents or legal guardians often ask General Practitioners (GPs) to screen their children for thyroid diseases.

Diagnostics of thyroid dysfunctions arefrequently based on the measurements of TSH serum concentration only. This is due to the fact that alog-linear relationship between TSH and FT4 and FT3 concentration is observed, thus abnormal TSH concentration suggests the possible occurrence of thyroid disorders [9,10]. In children, the level of TSH appears to be a stable and solid diagnostic parameter, however, its variability in laboratory tests has been observed [11]. On the other hand, there are some conditions in which the transitionally elevated TSH concentration is observed, with obesity being one of the well-documented examples [12]. Studies concerning the influence of other transient conditions (e.g., inflammations) on TSH secretion are divergent [13,14]. However, it was proven that the elevation of several proinflammatory cytokines may affect thyroid function tests, which mimic thyroid disorders in the absence of actual thyroid disease [15,16]. The best-known example of changes in the thyroid axis function, occurring also in children, is a non-thyroidal illness syndrome (NTIS) in critically ill patients. This phenomenon can resemble a response of healthy subjects to fasting [17]. Thyroid hormone inactivation with low T3 and high rT3 followed by suppressed TSH is the most frequently observed phenomenon in these patients. However, NTIS may also lead to elevated TSH. It was demonstrated that during the recovery phase from such diseases serum TSH level may be increased, but generally not higher than 10 mIU/L [8]. The slightly elevated TSH levels after acute infections were observed in adults [18]. The influence of NTIS on TSH concentration in children is well documented. In turn, TSH changes during the most common infections of the respiratory tract in children are observed in practice, however, data on their incidence and causes are scarce. This phenomenon was found because of the tendency to combine TSH tests with diagnostic tests made due to other reasons (e.g., infection), in order to minimize stressful situations for a child. If TSH serum concentration is outside the reference range, the diagnostic procedure used in patients with thyroid disorders isintroduced. Clinical observations indicate that in a considerable number of these patients, thyroid disease is not confirmed, and the results of the initial tests are–in fact–falsely positive.

Thus, the aim of the study was to assess the incidence of transient hyperthyrotropinemia in generally well children during acute infections of the respiratory system.

## 2. Patients and Methods

The research was approved by the Bioethical Committee at the Polish Mother’s Memorial Hospital Research Institute (PMMH-RI) in Lodz (approval code 69/2018).

The study included consecutive children who visited one GP (K.A.) in a single Primary Healthcare Centre due to mild or moderate respiratory tract infections over the period of one year. In every case, medical history, as well as signs and symptoms of the infection were recorded. Each child was physically examined, i.e., the throat and tonsils were assessed by inspection, the lungs were auscultated using a stethoscope and body temperature was checked using a non-contact thermometer. No chest X-rays were performed. Next, in every case, a complete blood count (CBC) test with differential was performed and C-reactive protein (CRP) in serum was measured. The tests were run on the next day after the visit at the physician’s (peripheral blood samples were collected between 8.00 and 10.00 a.m., after overnight fasting). Venous blood was obtained by venipuncture (needle gauge 19). At the same time, an additional blood sample was collected to assess TSH and FT4 concentrations. Moreover, during the visit, on the basis of the height and body mass measurements, the child′s nutritional status was evaluated, by assessment of the body mass index (BMI) value. Obese (BMI > +2.0 SD; n = 7) and undernourished (BMI < −2.0 SD; n = 2) children were excluded from the study, similarly to patients treated for thyroid diseases (n = 2). At the time of data collection, none of the children displayed typical signs and symptoms of thyroid disorders, e.g., goiter.

On the basis of the medical history, physical examination and the results of laboratory tests, the type of infection was diagnosed, based on the ICD-10 classification. The children were qualified into one of the following diagnostic groups: A88.0-enteroviral exanthematous fever (Boston exanthem), H66.0-suppurative otitis media, J00-acute nasopharyngitis (common cold), J02-streptococcal pharyngitis, J03-acute tonsilitis, J04-acute laryngitis, J06-acute nasopharyngitis, J18-pneumonia (unspecified), J20-acute bronchitis, J36-peritonsillar abscess.

It was noted whether the infection was accompanied by a fever over 38 °C and elevated lab inflammatory markers (Table 1).

Next, the parents were asked to bring the child for a check-up examination after ≥2 weeks after recovery. At the follow-up visit, the patient′s condition was evaluated again and a blood sample was taken between 8.00 and 10.00 a.m. in order to assess the same parameters: CBC, CRP, TSH and FT4. Thus, the blood samples were collected from the same patient at two consecutive time points: an initial visit during infection and a follow-up visit (which took place 2 weeks to 6 months after recovery). In each case where TSH was elevated the first time, the concentrations of thyroid peroxidase antibodies (a-TPO) and thyroglobulin antibodies (a-Tg) were assessed in the second blood sample.

Finally, 94 children (49 boys and 45 girls), aged 2.2–17.3 years, mean ±SD: 8.22 ± 3.98 years) were included in the study group. Among them, 76 children were qualified for the younger group: boys <12 years old and girls <11 years old, and 18 children for the older group: boys ≥12 years old and girls ≥11 years old.

The following parameters were considered significant inflammatory markers:

1. high CRP—according to World Health Organization recommendation-over 10 mg/L [19];
2. high white blood cell (WBC) count-above the reference range quoted on the test result, i.e., over 17.500 leukocytes/mm^3^ for children up to 5 years old, over 15.000 leukocytes/mm^3^ for children aged 6–12 years, and over 11.000 leukocytes/mm^3^ for children over 12 years;
3. increase in the proportion of lymphocytes (lymphocytosis)-above the reference range quoted on the test result, i.e., over 60% for children up to 5 years old, over 48% for children aged 6–12 years, and over 45% for children over 12 years;
4. increase in the proportion of neutrophils (neutrophilia)-above the reference range quoted on the test result, i.e., 51% for children up to 5 years old, over 59% for children aged 6–12 years, and over 55% for children over 12 years.

Concentrations of TSH and FT4 were measured by the electrochemiluminescent immunoassays (ECLIA) method with commercially available appropriate kits (Roche Diagnostic, Mannheim, Germany). Normal range values were as follows: for TSH: age-dependent ranges-1–7 years-0.7–5.97 mIU/L; 7–12 years-0.6–4.84 mIU/L; 12–18 years-0.51–4.4 mIU/L with inter-assay coefficients of variation (CVs) 1.3–1.8% and for FT4: age-dependent ranges-1–6 years-0.96–1.77 ng/dL; 6–11 years-0.97–1.67 ng/dL; 11–18 years-0.98–1.63 ng/dL with CVs 2.0–2.4%. All assays were performed as a part of a standardpatient care in the same laboratory cooperating with the GP clinic. The range of WBC, TSH and FT4 reference values were established on the basis of data from this laboratory.

The data were analyzed using Statistica 11.0 PL software (StatSoft, Inc., Tulsa, OK, USA). The continuous variables were expressed as mean ±SD for normally distributed variables. Shapiro–Wilk test was used to test the distribution of the variables. The differences between girls and boys were compared using chi^2^ test. Correlations were evaluated using the Pearson’s test. A one-way ANOVA was applied for statistical analysis with the subsequent use of a post-hoc test, in order to statistically assess differences between groups; Tukey’s test was selected because of the uneven amount of data in individual groups. To compare the frequency of cases with elevated TSH between the younger and the older group of children, Fisher’s exact test was used. *p* < 0.05 was accepted as statistically significant value.

## 3. Results

In the study group of the 94 children, 68 (72.3%) were diagnosed with an infection of the upper respiratory tract, while 26 (27.7%)-with an infection of the lower respiratory tract. The infection was accompanied by fever in over half (51.1%) of the examined children. Elevated CRP was observed in 31.9%, neutrophilia or elevated WBC were found in 23.4% and 29.8%, respectively while lymphocytosis-in 19.1% of the patients. Table 1 presents the results of the analyzed study group depending on the clinical diagnosis.

**Figure 1:**
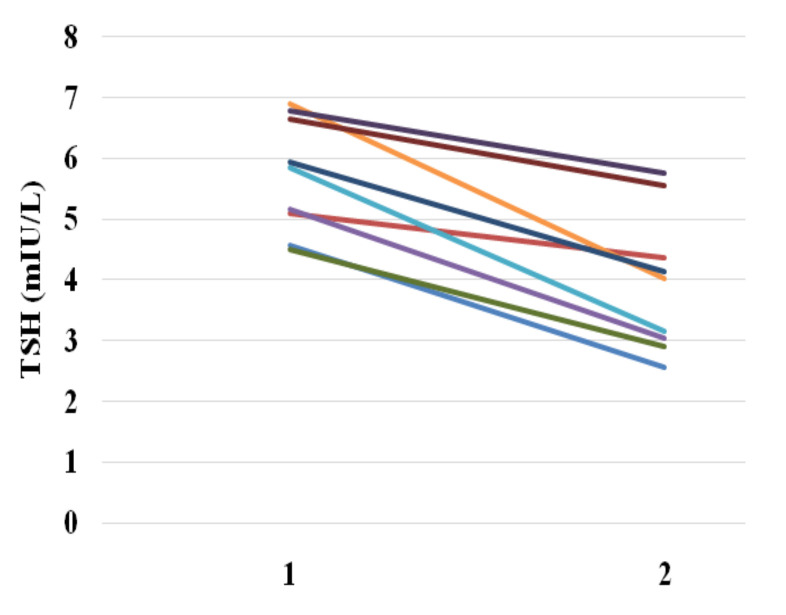
TSH concentration during infection (**1**) and during the check-up (**2**) in the group of children with elevated TSH concentration at the first examination (during infection).

In nine children from the studied group (9.6%), TSH concentration values exceeded the upper limit of the normal range, but none of them exceeded 7.0 mIU/L. In the check-up examination performed after recovery, elevated TSH values were not observed in any of the children (Figure 1). A detailed analysis of the cases in whichelevated TSH was found is presented in Table 2. Among children with elevated TSH found at the first time point (during infection), five patients had fever. In this subgroup (cases with elevated TSH concentration and fever), four children had elevated CRP, and among them, one had increased WBC and three neutrophilia, while in the fourth case, the CBC test result was normal. In the fifth child with fever, CRP and WBC were not elevated, but an increased number of neutrophils was observed. Three children did not have fever or elevation of any of the biochemical inflammatory markers.

In all of the analyzed cases, TSH concentration returned to normal in the check-up examination, performed after infection resolution. In none of the cases, FT4 concentration was reduced—the values ranged from 0.98 and 1.44 ng/dL, with the mean value of 1.25 ng/dL. An elevated a-TPO or a-Tg levels were not observed either.

In the whole group of the 94 examined children, TSH concentrations were analyzed with respect to the occurrence of inflammation symptoms mentioned above. Thus we compared TSH levels between the groups of children with and without fever, with normal and with elevated CRP, with normal and with elevated WBC, with normal and with an elevated count of neutrophils and with normal and with an elevated count of lymphocytes. Statistically significant differences between groups were not found, only in CRP-dependent analysis, the differences between groups were on the border of statistical significance (Table 3). A multivariance analysis confirmed that TSH level was not significantly influenced by any of the parameters in question (type of infection, fever, elevated value of CRP, WBC, elevated count of lymphocytes or neutrophils).

We also compared TSH and FT4 levels between the younger (prepubertal) and older (pubertal) groups of children. We found no differences between the groups in terms of the parameters mentioned above. We found that elevated TSH was more common in older children, but the relevance of this finding is uncertain due to the unequal number of children in the two groups (Table 4).

On the other hand, in the studied group of children, TSH concentration decreased in 61 out of 94 children (64.9%) in the check-up examination (after infection) regardless of its baseline value. Moreover, a reduction of more than 10% was observed in 37 children (39.4%). The mean values of TSH (±SD) were 2.93 ± 1.32 and 2.67 ± 1.05 mIU/L at the initial visit and the follow-up visit, respectively. The statistical analysis based on Wilcoxon′s signed-rank test showed significant differences between TSH levels at the initial visit and the follow-up visit(*p* = 0.007).

**Figure 2:**
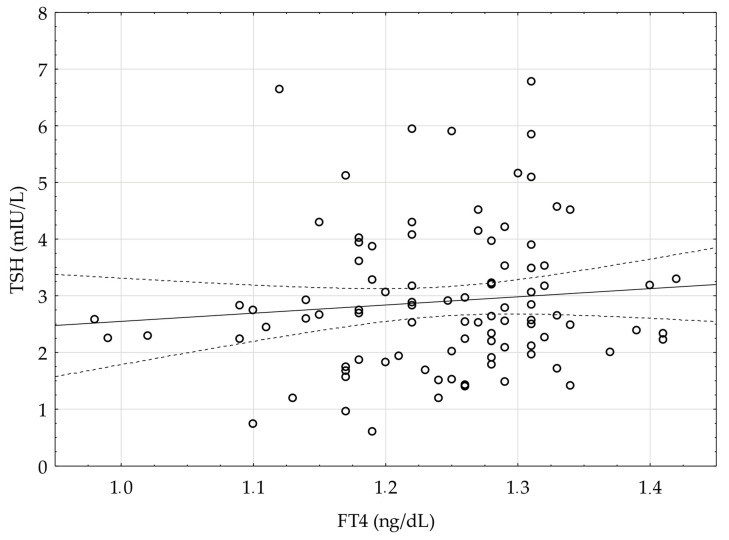
Correlation between TSH and FT4 concentration assessed in children during infection (Initial visit), r = +0.10, *p* > 0.05.

FT4 levels were within the normal range in all of the children and there was no significant correlation between TSH and FT4 levels, r = +0.10, *p* > 0.05(Figure 2). Moreover, FT4 concentration did not change between the initialand the follow-up visit.

## 4. Discussions

Every disease, including infections, especially in the acute phase, disturbs the balance between anabolic and catabolic processes. Thyroid hormones play an important role in maintaining homeostasis in response to environmental challenges. Beneficial adaptation includes decreasing energy consumption and stimulation of an immunological response, which are crucial to survival [16].

The host’s defense against infection involves the mechanisms of immunity and tolerance. While immunity promotes the elimination of pathogens, tolerance promotes adaptation to a given type of pathogen or to the intensity of the inflammatory response [20,21].

It is worth mentioning that there are two forms of adaptation activity. Type 1 allostasis occurs in diseases and conditions related to energy deficiency when the energy demand exceeds the supply and the stored energy resources. On the other hand, type 2 allostasis, occurs when the demand for energy does not exceed the potential supply, which is sufficient in relation to the needs [22,23]. Allostasis mediators involved in the acute phase reaction are proinflammatory cytokines. CRP is an acute-phase protein, whose synthesis in the liver is regulated by these cytokines. Its level is proportional to the concentration of inflammation mediators, which—in turn—positively correlates with the intensity of the inflammatory processes. CRP concentration exceeding 100 mg/L (often over 500 or 1000 mg/L) is usually found in serious bacterial infections. In the course of viral infections, usually normal or slightly elevated CRP concentrations are observed [24,25]. Therefore, we assessed CRP as one of the most important parameters indicating the type of infection and the phase response in our study.

The elevation of several proinflammatory cytokines may affect thyroid function tests. The acute phase of infection is mainly characterized by intensification of anterior pituitary hormone secretion and a peripheral inactivation of anabolic hormones [13]. One of the first alterations in acute illness is inhibition of type 1 deiodinase (D1) and type 2 deiodinase (D2) in peripheral tissues and subsequent impaired conversion of T4 to T3, leading to a decrease in serum and tissue T3 levels soon after the onset of acute illness [26]. D1 also deiodinates rT3, so the degradation is impaired and the levels of this inactive hormone rise simultaneously with the fall in T3 levels. Thus, TSH concentration temporarily increases in the first hours of the critical illness, which is followed by a temporary increase of T4 in the serum. Atthe same time, T3 concentration may already be reduced and reverse triiodothyronine (rT3) concentration may be elevated due to acute changes in the peripheral metabolism of thyroid hormone [27]. The scale of changes in T3 and rT3 concentration in the blood serum depends on the severity of the disease. This results in the NTIS [16]. In patients with mild to moderate NTIS, changes in T4 and TSH concentration are usually not observed, while patients suffering longer and from a more severe illness demonstrate low concentrations of T4 and TSH, as well [14].

In our study, in all the cases, we dealt with the acute form of common infection of the respiratory tract, which occurred in previously healthy, well-being children and was successfully cured in an outpatient clinic. Thus, the changes observed in NTIS, especially T3 and rT3 concentrations, were not a subject of our research. It is difficult to explain the reasons for the transient elevation of TSH levels observed in some children among our study group and the observed tendency to a higher TSH concentration during the infection period as compared to the period after recovery. We did not identify a significant correlation between increased TSH and individual, commonly available inflammatory markers, such as CRP, lymphocytosis and the presence of fever. Infection may trigger different reactions, depending on the kind of pathogen and the progress of infection. Thus, it may be difficult to clearly classify the observed abnormalities induces by infection into any of the two forms of adaptive activity. This may imply a more complex background of the phenomenon.

Temperature-induced changes of the thyroid or thyroid axis function are well known. One of them is the physiological increase in TSH concentration in response to exposure to cold, followed by an increase in the thyroid hormone synthesis, which stimulates metabolism [28].

However, the influence of high temperatures is ambiguous. The interesting data were provided by Oka et al. In their study, the evaluation of the influence of body temperature (BT), ranging from 37.5 to 40.5 °C, conducted in a group of 64 febrile patients, showed a relationship between BT and thyroid function. A negative correlation betweenTSH concentration and BT and a positive correlation between FT4 concentration and BT were observed. A detailed data analysis indicated that 8.2% of patients demonstrated an increased TSH concentration. This phenomenon was observed only for temperatures ranging from 37.5 to 38.5 °C and was not found in patients with higher temperatures [29].

Moreover, the experimental studies on the impact of temperature on thyroid function demonstrated that acute hyperthermia resulted in decreased blood flow to the thyroid gland and decreased secretion of FT4 and FT3, while TSH levels were not affected [30].

We also cannot disregard the significance of the loss of appetite during acute infection. The relationship between fasting and ghrelin, whose concentration correlates positively with TSH concentration, is well known. It is highly probable because the hormonal profile observed in the study group (increased TSH concentration during infection with no impact on FT4 concentration) is characteristic for the activity of ghrelin, whose elevated concentration directly inhibits T4 synthesis [31,32].

The highly complex processes, which take place in a living organism during acute infection may influence the thyroid axis at different levels and in an equally complex way. The concentrations of TSH and thyroid hormones are the result of all the processes that occur during the inflammatory reaction, which vary depending on the type and intensity of infection. Moreover, one of the key factors is the duration of infection, which is reflected in the severity and range of changes in the body functions. It should be remembered that even though changes in TSH concentration are usually not observed during NTIS, TSH transiently rises in the first hours of critical illness and during recovery [18,27].

Finding of TSH increase during mild or moderate upper respiratory tract infection may indirectly differentiate common infections in some children with coronavirus disease-19 (COVID-19), referring to the fact, the SARS-CoV-2 virus has a significant affinity to thyroid cells and may cause destructive thyroiditis and transient thyrotoxicosis accompanied by TSH suppression [33].

The limitations of our study, despite its prospective character, are differences in the time-lag between the infection onset and the exact moment when the patient first visited the GP and the patient′s blood was drawn. Moreover, we did not determine the patients’ hydration status with the application of the blood urea nitrogen/creatinine ratio assessment.

Our observation of the decreasing TSH concentrations after infection in nearly 2/3 of the children suggests that the secretion of this hormone during infection may result from simultaneous processes of stimulation (an increase in TSH concentration, typical of the first hours/days of infection, fasting, fever), as well as inhibition (D2 stimulation in the hypothalamus) of TSH secretion. The latter process occurs in parallel with the inhibition of the peripheral conversion of T4 to T3 [34].

Taking into account our results presented above, one should always remember that a diagnosis of thyroid dysfunctions made on the basis of a single TSH measurement only, though perhaps inexpensive and convenient, seems oversimplified and involves a considerable risk of obtaining falsely positive results. Our study demonstrated that acute infection is a condition that may lead to transient elevation of TSH, regardless of the level of inflammatory markers. Thus, the TSH level elevated in a child during or directly after an acute infection should be always re-assessed after the recovery.

## 5. Conclusions

1. Transient hyperthyrotropinemia was observed in about 10% of children suffering from an acute respiratory tract infection, regardless of its course, location and severity. The etiology for this finding is unclear.
2. If the test is performed during the infection and slightly elevated TSH concentration is noted, the TSH test should be repeated after recovery. Such an approach will allow many patients to avoid expensive diagnostic procedures and unnecessary implementation of treatment.

## Figures and Tables

**Table 1:** The clinical characteristics of the analyzed group of patients.

ICD-10		No.	Fever >38 °C	Elevated CRP	Elevated WBC	Neutrophilia	Lymphocytosis	Elevated TSH
A88.0	Enteroviral exanthematous fever (Boston exanthema)	1	1	0	1	0	0	0
H66.0	Suppurative otitis media	2	2	2	1	2	2	2
J00	Acute nasopharyngitis (cold)	7	1	0	1	1	1	1
J02	Streptococcal pharyngitis	25	19	10	7	7	7	3
J03	Acute tonsilitis	2	2	0	0	0	0	0
J04	Acute laryngitis	11	0	3	2	4	4	1
J06	Acute nasopharyngitis	19	5	4	1	5	7	1
J18	Pneumonia (unspecified)	5	2	3	1	2	1	0
J20	Acute bronchitis	21	9	7	5	6	2	1
J36	Peritonsillar abscess	1	1	1	1	1	0	0
Total		94 (100%)	48 (51.1%)	30 (31.9%)	20 (23.4%)	28 (29.8%)	18 (19.1%)	9 (9.6%)

**Table 2:** A detailed analysis of the cases where elevated thyroid stimulating hormone (TSH) was found.

					Initial Visit						Follow-Up Visit						
Gender	Age (years)	Type of Infection	ICD-10	Fever	CRP mg/L	WBC 10^9^/L	Neu %	Lym %	TSH mIU/L	FT4 ng/dL	Days between Initial and Follow-Up Visits	CRP mg/L	WBC 10^9^/L	Neu %	Lym %	TSH mIU/L	FT4 ng/dL
F	12	Upper resp. tract	H66	Yes	19.81 (↑)	13.9 (↑)	74.2 (↑)	13.9	4.57	1.33	30	<1	7.5	42	44.1	2.56	1.27
M	4	Upper resp. tract	H66	Yes	68.08 (↑)	10.1	52.5 (↑)	25.7	6.9	1.25	28	<1	8.06	37.2	49.8	4.02	1.19
M	11	Upper resp. tract	J00	No	<1	6.83	42.6	42.9	5.16	0.98	35	<1	6.44	50.1	36	3.04	1.21
F	13	Upper resp. tract	J02	Yes	7.03	5.34	59 (↑)	29	4.51	1.34	68	<1	4.13	49.9	39.7	2.91	1.29
F	14	Upper resp. tract	J02	Yes	10.51 (↑)	14.67(↑)	69.4(↑)	16.9	5.09	1.31	21	<1	6.85	43.9	45.0	4.36	1.23
M	6	Upper resp. tract	J02	No	3.26	11.33	32.1	55.5 (↑)	6.78	1.27	46	<1	5.77	56	33.8	5.77	1.33
F	3	Upper resp. tract	J04	No	<1	8.5	46.7	36.4	6.64	1.12	21	<1	7.91	29.2	58.3	5.56	1.18
F	11	Upper resp. tract	J06	No	1.04	9.3	44.9	43.9	5.95	1.22	37	<1	9.43	48	41.1	4.13	1.20
M	4	Lower resp. tract	J20	Yes	33.11 (↑)	4.21	35.9	35.9	5.85	1.44	15	<1	8.73	51	30.8	3.15	1.37

**Table 3:** The mean TSH levels (±SD) at the initial visit in children with and without fever, with and without elevated C-reactive protein (CRP), with and without elevated white blood cells (WBCs), with and without elevated neutrophils and with and without elevated lymphocytes.

	Normal	Elevated	*p* =
body temperature	2.93 ± 1.27	2.85 ± 1.27	0.76
CRP	2.74 ± 1.24	3.23 ± 1.27	0.08
WBC count	2.79 ± 1.18	3.23 ± 1.48	0.15
neutrophils count	2.83 ± 1.26	3.04 ± 1.27	0.48
lymphocytes count	2.90 ± 1.29	2.88 ± 1.23	0.98

**Table 4:** Characteristics of the analyzed group of children depending on the age (the younger group: boys <12 years old and girls <11 years old) and the older group: boys ≥12 years old and girls ≥11 years old).

	Younger Children n = 76	Older Children n = 18	*p* =
Girl/boys	32/44	13/5	
Age (years)	6.79 ±2.75	14.5 ±1.53	<0.000
TSH (mIU/L)	2.88 ± 1.29	2.95 ± 1.16	NS
FT4 (ng/mL)	1.24 ± 0.08	1.24 ± 0.11	NS
Elevated TSH n (%)	5 (6.6)	4 (22.2)	0.06

## Data Availability

The data presented in this study are available on request from the corresponding author.
